# Internal Loads, but Not External Loads and Fatigue, Are Similar in Young and Middle-Aged Resistance-Trained Males during High Volume Squatting Exercise †

**DOI:** 10.3390/jfmk3030045

**Published:** 2018-08-22

**Authors:** John F. T. Fernandes, Kevin L. Lamb, Craig Twist

**Affiliations:** 1 Department of Sport and Exercise Science, University of Chester, Chester CH1 4BJ, UK; 2 Higher Education Sport, University Centre Hartpury, Hartpury GL19 3BE, UK

**Keywords:** resistance training, training load, ageing

## Abstract

Little is known about the internal and external loads experienced during resistance exercise, or the subsequent fatigue-related response, across different age groups. This study compared the internal (heart rate, OMNI ratings of perceived exertion (RPE), session RPE) and external loads (peak velocity and power and volume load) during high volume squatting exercise (10 × 10 at 60% one-repetition maximum (1RM)) and the fatigue-related response (maximal voluntary contraction (MVC), voluntary activation (VA), resting doublet force, peak power, and blood lactate) in young (*n* = 9; age 22.3 ± 1.7 years) and middle-aged (*n* = 9; age 39.9 ± 6.2 years) resistance-trained males. All internal load variables and peak velocity illustrated unclear differences between groups during exercise. Peak power and volume load were likely higher in the young group compared to their middle-aged counterparts. The unclear differences in MVC, VA and blood lactate between groups after exercise were accompanied by very likely greater decrements in resting doublet force and peak power at 20 and 80% 1RM in the middle-aged group compared to the young group. These data indicate that internal load is not different between young and middle-aged resistance-trained males, though certain external load measures and the fatigue response are.

## 1. Introduction

Longitudinal resistance training induces muscle hypertrophy and increases in strength and power that are independent of age [1]. While such morphological adaptations have been noted in younger athletes [1], they have also been observed in older populations [1,2], for whom natural age-associated losses in muscle mass (sarcopenia) [3] and strength and power (dynapenia) [4] are expected. For the growing number of ‘middle-aged’ athletes (i.e., those 35 to 55 years) [4], resistance training can off-set or delay the effects of sarcopenia and dynapenia to maintain sporting performance [5]. 

To determine the efficacy of an athlete’s resistance training a coach must quantify the stress imposed on the athlete [6]. If the training load is insufficient then adaptation might not occur, whereas excessive or sudden increases in stress might result in injury or poor performance [7]. As such, practitioners should record markers of internal (i.e., the athlete’s individual responses, such as heart rate (HR) and ratings of perceived exertion (RPE)) and external (i.e., the work completed by the athlete, in terms of variables such as velocity, acceleration, and power output) loads to quantify the training stress. However, because of the numerous factors (e.g., movement velocity, rest times, relative intensity, volume load) that can invoke a resistance training adaptation, there is no consensus regarding the best method to monitor resistance training load [6]. 

There is evidence to indicate that internal load variables might differ between age groups when exercising at the same relative external load. For example, higher absolute heart rates [8] and blood lactate concentration [9], and lower [10], higher [11] and similar [12] RPEs have been noted in young (~21 to 28 years) compared to older (~57 to 84 years) males during resistance exercise. These findings are despite observations of no differences in absolute or relative heart rate [13] or blood lactate and RPE [14] at the point of muscular failure between young (~21 to 28 years) and older (~48 to 67 years) males. Furthermore, to the authors’ knowledge, no study has yet compared the external load between age groups during resistance training exercise despite external load being the primary driver of resistance training adaptations [15]. A limitation of focusing on external or internal load in isolation is that they might not be able to reflect the internal load for a given external load. Therefore, calculating an internal to external load ratio might negate the poor sensitivity and inter-individual variability of individual training load metrics [16]. The use of external load markers in isolation demonstrates a limited relationship with measures of endurance capacity (velocity at lactate threshold, velocity at 4 mmol·L^−1^ and VO_2max_), whereas the external to internal load ratios exhibit moderate to large correlations (*r* = 0.41 to 0.69) [16,17]. These data might suggest that the integration of internal and external load is a more sensitive measure of overall training load. However, the application to resistance type exercise is yet to be explored.

The subsequent fatigue (i.e., inability to maintain the expected force or power output) [18] response to resistance exercise between age groups is unclear [19,20]. Two recent meta-analyses concluded that ageing is associated with less fatigue after isometric contractions, but not dynamic contractions, when assessed in terms of force production during maximal voluntary contractions [19,20]. When velocity and power are used as markers of fatigue, older (~64 to 75 years) males experience greater fatigue than their young (~27 years) males during knee extension [21,22,23] but not during sit-to-stand exercise [20,23]. It has been suggested that the age-related slowing of the muscle is responsible for the greater fatigue during knee extension exercise [21,23], whilst the group similarity in fatigue during sit-to-stand exercise was attributed to task specificity; both groups would typically perform sit-to-stand tasks but not knee extension movements [23]. However, the findings of these studies might not be applicable to the middle-aged male who regularly resistance exercises and plays sports because single-jointed knee extension and sit-to-stand movements are not applicable to the multi-jointed compound movements involved in such activities. A study that quantifies the fatigue response from an ecologically valid resistance training protocol would therefore be particularly beneficial to the resistance trained middle-aged male.

Another plausible explanation for the differences in the fatigue response between age groups might be sought from the internal and external loads experienced during exercise. That is, greater fatigue might be an artefact of a higher internal or external load during exercise of the same relative load. Resistance training protocols with a large amount of work performed are subject to greater decrements in isometric force [24,25]. However, no study has investigated the relationship between internal load and post-exercise decrements in muscle function. Moreover, despite the efforts of two studies [9,13], the age-related research has focused solely on those aged over ~60 years, none of whom were resistance trained. Thus, the stress imposed during resistance exercise in middle-aged males, compared to younger males, is unknown. The findings from a study that quantifies the internal and external load in middle-aged (35 to 55 years) males would be particularly useful for middle-age men who seek to monitor their resistance training. Consequently, the primary aim of this study was to quantify the internal and external loads experienced in lower-limb resistance exercise in young and middle-aged males who regularly resistance train and to determine the fatigue responses to such exercise. A further aim was to determine the relationship between internal and external load with post-exercise decrements in muscle function.

## 2. Materials and Methods

### 2.1. Participants

Nine young (21 to 25 years) and nine middle-aged (35 to 54 years) resistance trained males were recruited for this study from the University population, local gymnasia, and sports teams using convenience sampling. Thirty-five years was selected as the lower boundary for the middle-aged group because it is the entry age for ‘Masters’ athletes (see British Masters Athletic Federation and World Masters Athletics). As age-related studies typically use older groups (60 years and over), 55 was selected as the upper-limit for the middle-aged group. All participants took part in sport (i.e., team sports, racket sports and endurance type sports) for a minimum of two years (4.1 ± 1.3 and 18.0 ± 5.6 years for the young and middle-aged groups, respectively) and had a minimum of two years’ resistance training experience and regularly used squats as part of their resistance training programmes. Participants completed a pre-test health questionnaire and provided written consent for the study, which was approved by the Ethics Committee of the Faculty of Life Sciences at the University of Chester. 

### 2.2. Design

**Figure 1:**
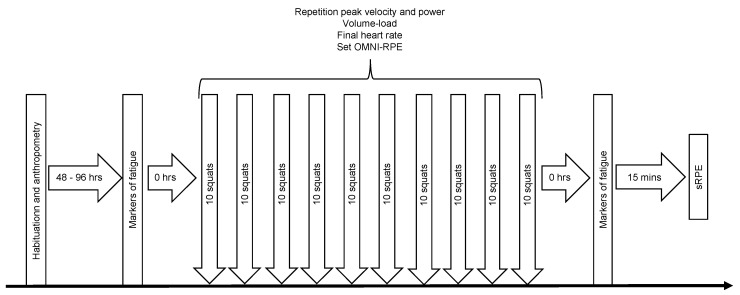
Schematic of study design.

The study used a mixed factorial design that required attendance at the strength and conditioning laboratory on two separate occasions (Figure 1). Participants were instructed not to consume any ergogenic supplements (for example, caffeine) on each occasion and to refrain from heavy exercise between visits. On the first occasion, they provided biometric data (stature, body mass and skinfold thicknesses for the assessment of body composition), an estimate of back squat one-repetition maximum (1RM), and were habituated with the measurements of lower limb peak power, maximal voluntary contraction (MVC), and voluntary activation (VA) during isometric knee extension. Participants were considered ‘habituated’ when they could complete three consecutive repetitions that produced peak powers or torque values each within 10% [4,26]. On returning to the laboratory 2–4 days later, they provided measurements of peak power during squats at 20 and 80% 1RM, MVC, VA and blood lactate before and after an exercise bout comprising 10 × 10 squats at 60% 1RM [27]. During the exercise bout, bar peak velocity and power were recorded for each repetition, and heart rate and RPE were recorded at the end of each set. Session RPE (sRPE) was recorded 15 minutes after the squatting exercise bout. Participants were not provided with any feedback during the study that might have influenced their sRPE. 

### 2.3. Procedures

#### 2.3.1. Biometric Measures

Body mass and stature were determined using digital scales (Seca 813, Hamburg, Germany) and a wall-mounted stadiometer (Harpenden, Holtainm Crymych, Dyfed, UK). Body composition was assessed via skinfold thickness measurements (Harpenden, British Indicators, Burgess Hill, UK) taken at the tricep, axilla, abdominal, suprailliac, chest, subscapular, and mid-thigh incorporated into the equation of Jackson and Pollock [28] for predicting body density (Db). Body fat percentage (%BF) was derived from the equation [29]: %BF = [(5.21/Db) − 4.78] × 100. From this, the quantities (kg) of fat-mass (FM) and fat-free mass (FFM) were also derived.

#### 2.3.2. Maximal Strength Testing

To avoid the risk associated with maximal strength testing, one repetition maximum (1RM) for squat exercise was predicted using a three-repetition maximum (3RM) protocol. In brief, participants performed 8–10 repetitions with 50% of their estimated 1RM, followed by 3–5 repetitions at 85% of estimated 1RM. The load was then set at the approximate 1RM and the participants performed one repetition. The load was progressively increased until the participant could no longer perform a complete repetition. The final load lifted was used with the following equation [30] to estimate 1RM squat load:

1RM = (100 × load lifted)/(48.8 + (53.8 × 2.71828^−0.075×repetitions^)) (1)

The above equation has been reported to yield accurate 1RM predictions (*r* = 0.969, 0.02% different from direct 1RM) [31].

#### 2.3.3. Assessment of Peak Power during Back Squat

Peak power was assessed at loads corresponding to 20% and 80% 1RM during back squat exercise using a rotary encoder (FitroDyne, Fitronic, Bratislava, Slovakia) attached via a nylon cord directly under a Smith machine bar (Perform Better, Leicester, UK). As the FitroDyne measures rate of displacement and assumes that the nylon cord is moving in a vertical plane, a Smith machine was used to prevent deviation from this plane and decrease measurement error. The FitroDyne has been shown to produce reliable intra-day measures of peak power (coefficient of variation = 3.9–4.9%) at the selected loads [26].

With the bar positioned across the shoulders, participants squatted until their hips were below the knee joint and then ascended as rapidly as possible until their knees were at full extension. A bench was employed to ensure that they attained the same depth and range of motion on each repetition. Three repetitions at each load were performed with self-selected rest intervals that ranged from 30 to 90 s [26]. Rest times were self-selected, as lighter loads (20% 1RM) did not require the same recovery time. Peak velocity was recorded from which peak power was calculated as (load × velocity × 9.8)/100. The load order was randomised for each participant to negate possible ordering effects. 

#### 2.3.4. Assessment of Maximal Voluntary Contraction and Voluntary Activation

Before undertaking the MVC and VA assessments, participants performed a warm-up comprising five minutes of cycling at 100 W (Lode, Corival, Groningen, The Netherlands). A dynamometer (Biodex, Multi-joint system 3, Biodex Medical, New York, NY, USA) was used to measure isometric force of the participant’s dominant knee extensors at 80° knee flexion. To prevent extraneous body movements, Velcro straps were applied tightly across the chest and thigh. Participants were provided with strong verbal encouragement and real-time feedback via the PC monitor.

The knee extensors were electrically stimulated (5 s with two 100 Hz single square impulses (doublet); Digitimer, D57, Hertfordshire, UK) using two 5 × 13 cm moistened surface electrodes (Axelgaard Manufacturing Co., Ltd., Fallbrook, CA, USA); one placed distally over the quadriceps, and the other proximally over the upper quadriceps. During optimisation, the amplitude of a doublet was progressively increased, starting at 50 amps, until a point where no further increases in intensity resulted in an increase in resting doublet force. Initially, a 230 volt electrically evoked doublet (set 20% above the value required to evoke a resting muscle doublet of maximum amplitude) was applied to the resting muscle (resting doublet) at 1 s. The resting doublet was used to elucidate any peripheral alterations that might have occurred as a result of the squatting protocol. Participants then performed a 4 s MVC before a doublet which was applied at the isometric plateau (superimposed doublet). The MVC was taken as the average force over 50 ms (AcqKnowledge 3 software, Biopac Systems, Cambridge, MA, USA) before the superimposed doublet was applied. VA was calculated according to the interpolated twitch ratio using the equation

VA (%) = [1 − (size of interpolated doublet/size of resting doublet)] × 100(2)

A similar procedure has been deemed a reliable method (coefficient of variation = 3.38%) for assessing VA [32].

#### 2.3.5. High Volume Squat Exercise

The exercise protocol consisted of 10 sets of 10 repetitions of squat exercise at a load corresponding to 60% 1RM with 120 s rest between sets [27]. For each repetition participants descended for 3 s until their hips were below the knee joint and then ascended as rapidly as possible until their knees reached full extension. A bench was employed to standardise the depth of each repetition. The FitroDyne was used to calculate power for each repetition in the manner outlined above. Mean peak velocity and power over the sets was used to determine the relationship between external load during the exercise and alterations in the markers of fatigue. Volume load was calculated as the 60% 1RM load multiplied by 100.

#### 2.3.6. Assessment of Heart Rate

Heart rate (HR) was recorded at rest and at the end of each set using a chest strap (Polar Electro, Polar Beat, Oy, Finland). 

#### 2.3.7. Assessment of Perceived Exertion

At the end of each set, participants provided a global indication of their perceived exertion using the OMNI-RPE scale [33], which ranges from 0 to 10, 0 indicating ‘extremely easy’ and 10 corresponding to ‘extremely hard’. Previously, participants were provided with detailed instructions on how to rate their exertion. The OMNI-RPE scale is deemed a valid measure of perceived exertion during resistance exercise [33]. Additionally, sRPE was recorded 15 minutes after the completion of exercise. Participants were asked ‘How intense was your session?’ and ranked their exertion on a 1 to 10 scale, where 1 indicates ‘really easy’ and 10 indicates ‘maximal’. This method has been deemed a valid [34] and reliable [35] indicator of resistance exercise intensity.

#### 2.3.8. Assessment of Blood Lactate Concentration

Blood was obtained before and immediately after the exercise bout from a finger-tip capillary sample and analysed for lactate concentration using a Lactate Pro analyser (Arkray, Kyoto, Japan). The Lactate Pro has been deemed a reliable marker of blood lactate concentrations (coefficient of variation: 2.8 to 5.0%) [36].

#### 2.3.9. External to Internal Load Ratios

External load was quantified using mean peak velocity and power over the 10 sets of exercise and total volume load. Internal load was quantified using measures of mean heart rate and OMNI-RPE. External load was divided by each measurement of internal load to calculate the external to internal load ratio for the exercise protocol [16].

### 2.4. Statistical Analysis

Changes in dependent variables were examined using Bayesian analysis that employed the effect size (ES) with associated 90% confidence intervals (CI) [37]. This approach allowed for a more practical and meaningful explanation of the data that is deemed more useful to the coach and athlete when determining the magnitude of change in key measures of exercise performance during and after high-volume squatting exercise. Thresholds for the magnitude of the observed change for each variable were determined as the within-participant standard deviation in that variable × 0.2, 0.6 and 1.2 for a small, moderate and large effect, respectively [38]. Threshold probabilities for a meaningful effect based on the 90% CI were: <0.5% most unlikely, 0.5–5% very unlikely, 5–25% unlikely, 25–75% possibly, 75–95% likely, 95–99.5% very likely, >99.5% most likely. Effects with CI across a likely small positive or negative change were classified as unclear [37]. The rate of change of peak velocity and power, HR and OMNI-RPE during exercise was expressed as the slope of the regression line (beta coefficient) [39] of the dependent variables over the ten sets. A post hoc power calculation indicated that a sample size of 12 to 14 was needed to detect the changes in muscle function observed in the current study. All calculations were completed using predesigned spreadsheets (www.sportsci.org). Data are presented as ES, lower CI and upper CI. Pearson correlations were employed to quantify the association between the markers of internal and external load and the decrements in muscle function after squat exercise. The following scales were used to interpret the magnitude of the correlations: <0.1 trivial, 0.1–0.3 small, 0.31–0.5 moderate, 0.51–0.7 large, 0.71–0.9 very large, >0.9 nearly perfect [38]. Threshold probabilities for a meaningful effect based on the 90% CL were calculated using a predesigned spreadsheet [40]. 

## 3. Results

### 3.1. Biometric Measures and Training History

Age and sum of skinfolds were most likely and likely higher, respectively, in the middle-aged group compared to the young group (Table 1). Differences in fat mass and body fat percentage between the young and middle-aged groups were very likely between groups while mass and squat 1RM were unclear.

### 3.2. Internal Load Measures

**Figure 2:**
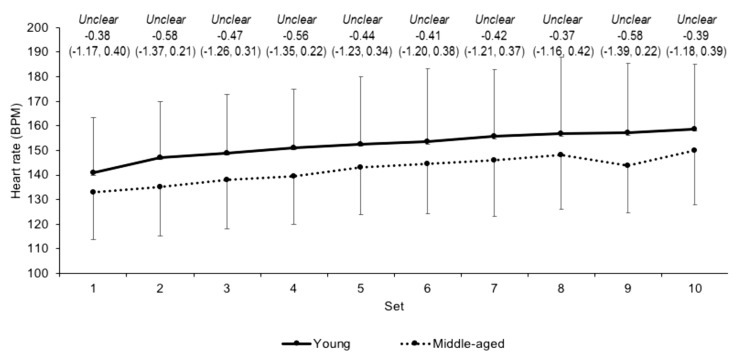
Absolute heart rate scores (mean ± SD) across each set for young and middle-aged groups. Qualitative descriptor, effect size, and upper and lower 90% confidence intervals are noted above.

**Figure 3:**
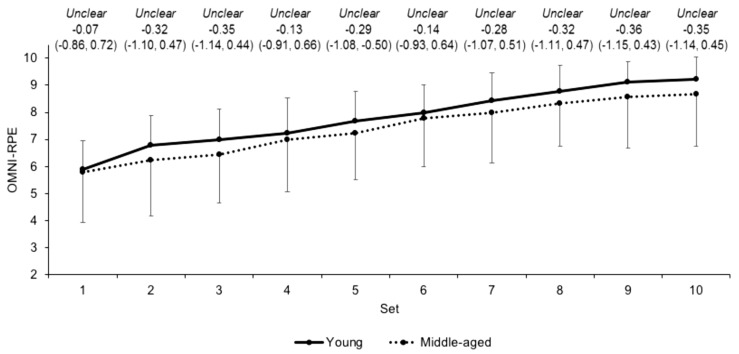
OMNI-RPE scores (mean ± SD) across each set for young and middle-aged groups. Qualitative descriptor, effect size, and upper and lower 90% confidence intervals are noted above.

Differences in heart rate (Figure 2) and OMNI-RPE (Figure 3) were unclear between the young and middle-aged groups over the sets. Differences in mean sRPE (7.7 ± 1.2 and 7.8 ± 1.3 for the young and middle-aged groups, respectively) were also unclear (ES 0.09, CI −0.72, 0.89). The rate of change for HR over the sets was unclear (ES 0.17, CI −0.63, 0.98) between young (*b* = 1.72 ± 0.96) and middle-aged (*b* = 1.91 ± 1.13) groups, as was the beta coefficient (*b* = 0.36 ± 0.09 and 0.34 ± 0.17, respectively) for OMNI-RPE (ES 0.17, CI −0.98, 0.65).

### 3.3. External Load Measures

**Figure 4:**
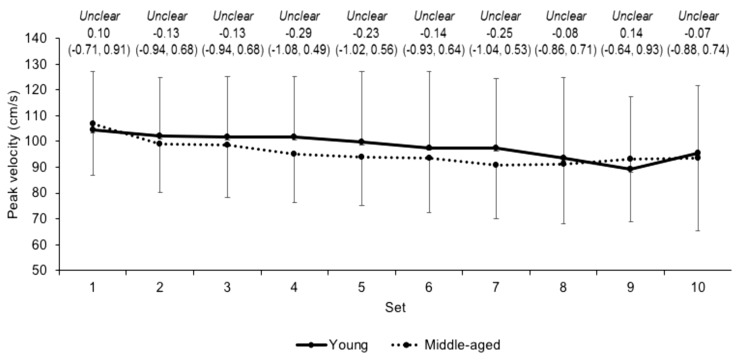
Peak velocity (mean ± SD) across each set in young and middle-aged groups. Qualitative descriptor, effect size, and upper and lower 90% confidence intervals are noted above.

**Figure 5:**
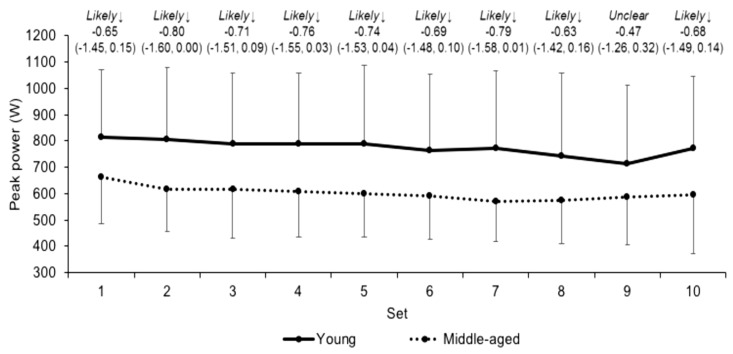
Peak power (mean ± SD) across each set in young and middle-aged groups. Qualitative descriptor, effect size, and upper and lower 90% confidence intervals are noted above. ↑ and ↓ denote higher and lower, respectively, in the middle-aged than young group.

Differences in peak velocity over the sets between the young and middle-aged groups were unclear (Figure 4). Differences in peak power over the sets were likely moderate (Figure 5) between the groups, except for set 9 where differences were unclear. The unclear (ES −0.12, CI −0.92, 0.69) differences in mean peak velocity for the young (97.9 ± 24.9 cm/s) and middle-aged (95.2 ± 19.7 cm/s) groups over the sets was accompanied by likely moderate differences in mean peak power (ES −0.71, CI −1.53, 0.10; 770.4 ± 278.0 and 603.2 ± 162.6 W for the young and middle-aged groups, respectively). Moreover, there was a likely moderate (ES −0.90, CI −1.70, −0.09) higher volume load in young (7898.2 ± 1560.0 kg) group compared to the middle-aged (6556.9 ± 1349.1 kg) group. Differences in mean beta coefficients for velocity and power across the sets were unclear (ES 0.31, CI −0.50, 1.11 and ES 0.31, CI −0.51, 1.10, respectively) between young (*b* = −1.7 ± 2.8 and −11.8 ± 20.5, respectively) and middle-aged (*b* = −0.9 ± 2.6 and −5.9 ± 18.2, respectively) groups. 

### 3.4. External to Internal Load Ratios

Differences in the external to internal load ratios between the groups were all unclear (Table 2).

### 3.5. Markers of Fatigue after Squatting Exercise

At Pre, the likely moderate differences in MVC (ES −0.80, CI −1.61, 0.01) and resting doublet force (ES −0.96 CI −1.77, 0.14) between the groups were accompanied by very likely moderate differences in 20 (ES −1.03, CI −1.84, −0.22) and 80% (ES −1.03, CI −1.84, −0.21) 1RM peak power. Differences in VA (ES 0.03, CI −0.77, 0.84) and blood lactate (ES −0.53, CI −1.34, 0.28) were unclear between the groups at Pre. The high volume squatting exercise was effective in causing decreases in markers of fatigue that were very likely for MVC (ES −0.96, CI −1.52, −0.39) and VA (ES −1.06, CI −1.63, −0.48), most likely for resting doublet force (ES −1.35, CI −1.92, −0.79) and likely for 80% 1RM peak power (ES −0.57, CI −1.13, 0.00). Alterations in 20% 1RM peak power were unclear compared to Pre (ES −0.24, CI −0.80, 0.33). Blood lactate concentration had most likely (ES 2.38, CI 1.82, 2.95) increases after the squatting exercise. After the squatting exercise the middle-aged group showed very likely greater decrements in resting doublet force and peak power at 20 and 80% 1RM than the young group (Table 3). Between-group differences after the exercise protocol were unclear for MVC, VA, and blood lactate.

### 3.6. Relationship between Internal and External Load Markers with Fatigue

Only mean HR and OMNI-RPE were related to the muscle function markers for the internal load variables (Table 4). That is, mean HR was likely (*r* = 0.45, CI 0.06, 0.72) and very likely (*r* = 0.50, CI 0.13, 0.75) correlated with decrements in MVC and peak power at 80% 1RM, respectively, while OMNI-RPE was likely correlated with alterations in peak power at 20 (*r* = 0.36, CI −0.05, 0.66) and 80% 1RM (*r* = 0.32, CI −0.09, 0.64). For external markers of load, changes in mean peak power were likely correlated (*r* = 0.35 to 0.43) with all decrements in muscle function. Similarly, a higher volume load during the protocol was very likely related to changes in the muscle function markers (*r* = 0.50 to 0.59). 

## 4. Discussion

To our knowledge, this is the first study to compare internal and external load variables, and fatigue response from squatting exercise, in resistance trained young and middle-aged males. These data indicate that the internal load during squatting exercise at the same relative intensity is not different in these groups, though certain measures of external load (i.e., volume load and peak power) are. Moreover, when compared to younger males, middle-aged males can expect greater decrements in peak power after squatting exercise, which appear to be related to certain internal (HR and OMNI-RPE) and external (peak power and volume load) load measures.

This study recorded unclear differences in HR and the HR rate of change during the resistance exercise between the two age groups. These data contrast to previously observed differences in HR between young and older physically active men during isometric knee extension exercise [8], but reaffirm no difference in HR between younger and older males during leg press exercise [13]. Similarly, the unclear differences observed in OMNI-RPE and the OMNI-RPE rate of change over the resistance exercise protocol are supported by previous data [14] but oppose previous findings in young and older males [10,11]. The similar internal responses between groups in the current study might reflect similar alterations in vagal tone and motor command [8,41] during resistance exercise in young and middle-aged males who regularly resistance train. sRPE demonstrated no differences between groups after the exercise, which is surprising given that sRPE is related to the volume load [42] that was moderately higher in the young group. sRPE appears to monitor the participant’s perception of the exercise in the context of the physical and psychological state [43], which indicates that, holistically, the resistance trained young and middle-aged males perceived the exercise similarly. For blood lactate concentrations, unclear differences between groups after resistance exercise emerged. Though higher blood lactate concentrations have been observed in younger compared to older males [9], the similarities in the current study might suggest a similar reliance on glycolytic pathways during the squatting exercise in the two groups. The current study also observed no differences in any external to internal load ratios, which would indicate that the internal response for a given external load is similar between young and middle-aged males during squatting exercise. Collectively, these data suggest that internal load markers in young and middle-aged resistance trained males are similar during high volume squatting exercise at the same relative load.

Given that young resistance trained males can produce higher velocities than middle-aged males [4] it is perhaps surprising that differences in the peak velocity between groups during the exercise protocol were unclear. However, differences in velocity during exercise between age groups might only be present during less familiar movements, albeit 60% 1RM for squat demonstrated the lowest differences between groups (ES = 1.0) [4]. Also, the repeated squatting in this study, compared to single repetitions performed previously [4], might have been subject to pacing in order to prevent premature fatigue. A further explanation for the differences in velocity during exercise between age groups might come from the participants’ familiarity with the movement. For example, Petrella and colleagues [23] noted greater fatigability and lower velocity in older adults (~64 years) compared to their young (~27 years) counterparts during knee extension exercise, but no differences were present during explosive sit-to-stand exercise. No difference in sit-to-stand exercise was attributed to familiarity with that movement in both groups, i.e., they would perform sit-to-stand movements in their daily routines whereas the older group were not familiar with knee extension exercise [23]. Given that all participants regularly squatted as part of their resistance programmes, this would explain no difference in peak velocity between groups in the current study. Over the exercise protocol, peak power was moderately higher in the young group compared to the middle-aged group while the rate of change in peak power was unclear between groups. This supports previous observations of lower power output and similar fatigability during explosive sit-to-stand exercise [23]. Petrella and colleagues [23] noted that differences in power between ages were driven by differences in velocity during exercise, yet the current study observed no differences in velocity. That power is the product of the velocity and force (i.e., the load) would indicate that the differences in peak power in the current study are due to the higher volume load performed by the young males. That is, the differences in power between young and middle-aged resistance-trained males during the exercise are a consequence of differences in force (i.e., the volume load) and not velocity as suggested by Petrella et al. [23] in young and old males. Accordingly, this study indicates that peak power, but not peak velocity, is higher in young compared to middle-aged resistance trained males during high volume squatting exercise.

Reductions in muscle function immediately after the squatting exercise are indicative of fatigue (i.e., inability to maintain the expected force or power output) [18]. Lower VA after the squatting exercise suggests that impairments in force and peak power were influenced by a reduction in drive to the muscle caused by neural impairments and a reduction in excitability to the alpha motor-neuron [33,44,45]. In addition, the lower resting doublet after exercise indicates peripheral alterations, that is, a disruption of sarcomeres and impaired excitation-contraction coupling and the accumulation of fatigue-related metabolites [46,47] might have also contributed to the reductions in MVC and peak power at 80% 1RM after the squatting. After exercise, resting doublet force and peak power at 20 and 80% 1RM had very likely greater decrements in the middle-aged group compared to the young group, where differences in MVC and VA were unclear. Greater fatigue in older populations after isoinertial compared to isometric actions are well supported [19,20] and may reflect an elevated energy cost of contraction [48] and impairments in cross-bridge cycling [21] with age. The greater decrements in resting doublet force in the middle-aged males contrast to the similar reductions between age groups after knee extension exercise reported by Dalton and colleagues [21] and are indicative of greater peripheral alterations (i.e., disruption of sarcomeres and impaired excitation-contraction coupling) [46,47] after high volume exercise. The unclear differences between groups in VA are similar to those previously reported by Dalton and colleagues [21] and suggest comparable central alterations after high volume exercise. As such, middle-aged trained males can expect a similar isometric, but not peak power, fatigue response after high volume squatting exercise.

Mean HR during exercise was moderately correlated with decrements in MVC and 80% 1RM peak power (*r* = 0.45 and 0.50, respectively). It is unknown why a greater cardiovascular load during squatting exercise might result in larger impairments in MVC and peak power at high external loads. Previous work by Rezk and colleagues [49] noted that elevated HR, albeit after resistance exercise, was associated with a cardiac sympathetic activation and parasympathetic deactivation. Like Rezk et al. [49], the higher HR in the current study are likely to driven by alterations in cardiac sympathetic and parasympathetic activity, which aim to increase oxygen delivery to the working musculature. OMNI-RPE was moderately associated with decrements in peak power at both 20 and 80% 1RM (*r* = 0.36 and 0.32, respectively). It is suggested that perception of effort reflects central motor command to the muscles [41]. Moreover, an increase in central motor command might seek to augment muscle activation in order to lift the load when the muscle is fatiguing [41]. Thus, it is understandable that an elevated OMNI-RPE would be associated with reductions in post-exercise fatigue markers. These data indicate a dose-response relationship between HR and OMNI-RPE during high volume resistance exercise and post-exercise decrements in muscle functional markers. Practitioners should be cognisant of the relationship between higher HRs and OMNI-RPEs with post-exercise decrements in muscle function. This study also reported those with a higher volume load were subject to greater impairments in MVC and peak power at 20 and 80% 1RM (*r* = 0.59, 0.55 and 0.50, respectively). These data are similar to previous observations of greater reductions in MVC after lower-limb resistance protocols with a higher amount of work performed [24,25]. The moderate correlations with average peak power during exercise and post-exercise reductions in MVC and peak power at 20 and 80% 1RM are the first of their kind. Like the suggestions of Brandon et al. [24] and Howatson et al. [25], these reductions in MVC might be owing to metabolic (i.e., increased use of the glycolytic pathway, which is indirectly supported by the higher post-exercise blood lactate) and peripheral alterations (i.e., impaired excitation-contraction coupling, demonstrated by the reduction in resting doublet scores after exercise). The relationships between external load (volume load and mean peak power) with post-exercise decrements in peak power during back squat are novel and indicate that a dose-response relationship exists between these variables. Importantly, these data suggest that the applied practitioner can monitor volume-load and mean peak power during resistance exercise should they need to be mindful of the post-exercise impairments in muscle function after lower-limb exercise.

## 5. Conclusions

This study examined the load (internal and external) and fatigue response in young and middle-aged males after high volume squatting exercise. These data indicate that internal load is not different between young and middle-aged resistance-trained males during squatting exercise, though certain external load measures (peak power and volume load) are. Practically, these findings suggest that internal, but not external, load can be used to monitor high volume resistance training in a like manner between these age groups. Moreover, high-volume squatting exercise impairs peak power at low and high external loads to a greater extent than isometric force in middle-aged males compared to their young counterparts. The applied practitioner should be mindful of these reductions in peak power in middle-aged males and programme lower-body resistance training accordingly. The correlations observed in this study indicate that certain internal (HR and OMNI-RPE) and external (mean peak power and volume-load) load are positively related to the post-exercise decrements in muscle function. As such, it is suggested that applied practitioners monitor these variables when post-exercise decrements in muscle function are undesirable.

## Figures and Tables

**Table 1:** Biometric characteristics (mean ± SD) of the young and middle-aged groups. Qualitative descriptor, effect size, and upper and lower 90% confidence intervals are noted in the effect size column.

Characteristic	Young (*n* = 9)	Middle-Aged (*n* = 9)	Effect Size
Age (y)	22.3 ± 1.7	39.9 ± 6.2	Most likely ↑
			3.70 (2.87, 4.53)
Mass (kg)	82.0 ± 9.0	79.1 ± 10.3	Unclear
			0.29 (−1.10, 0.52)
Fat-free mass (kg)	71.4 ± 7.9	63.9 ± 6.5	Very likely ↓
			−1.02 (−1.83, −0.22)
Fat-mass (kg)	10.5 ± 4.5	15.2 ± 5.7	Likely ↑
			0.89 (0.09, 1.70)
Body fat (%)	12.8 ± 4.7	18.8 ± 5.8	Very likely ↑
			1.13 (0.32, 1.94)
Sum of skinfolds (mm)	82.3 ± 24.6	102.4 ± 31.9	Likely ↑
			0.69 (−0.12, 1.50)
Squat 1RM (kg)	130.8 ± 26.8	109.3 ± 22.5	Unclear
			−0.85 (−1.65, −0.04)

↑ and ↓ denote higher and lower, respectively, in the middle-aged than young group.

**Table 2:** The external to internal load ratio during the exercise protocol in the young and middle-aged groups. Qualitative descriptor, effect size, and upper and lower 90% confidence intervals are noted in the effect size column.

Load Ratio	Young	Middle-Aged	Effect Size
HR: peak velocity	0.7 ± 0.2	0.7 ± 0.2	Unclear
			0.10 (−0.71, 0.90)
HR:peak power	5.2 ± 2.0	4.3 ± 1.3	Unclear
			−0.51 (−1.32, 0.30)
HR:volume load	52.2 ± 11.8	47.0 ± 13.0	Unclear
			−0.41 (−1.22, 0.39)
OMNI-RPE: peak velocity	12.6 ± 3.3	13.3 ± 2.7	Unclear
			0.21 (−0.60, 1.01)
OMNI-RPE: peak power	99.5 ± 36.6	84.8 ± 23.1	Unclear
			−0.47 (−1.28, 0.34)
OMNI-RPE: volume load	1030.2 ± 244.6	968.5 ± 451.2	Unclear
			−0.14 (−0.95, 0.68)

**Table 3:** Markers of fatigue (mean ± SD) in after squatting exercise in young and middle-aged males. Qualitative descriptor, effect size, and upper and lower 90% confidence intervals are noted in the effect size column.

Fatigue Indicators	Group	Pre	Post	Comparison
MVC (N/m)	Young	265.7 ± 95.8	179.2 ± 60.7	Unclear
	Middle-aged	199.1 ± 63.3	144.9 ± 55.4	−0.56 (−1.37, 0.25)
VA (%)	Young	93.4 ± 5.8	85.3 ± 9.4	Unclear
	Middle-aged	93.6 ± 5.6	82.9 ± 12.9	−0.20 (−1.00, 0.61)
Resting doublet (N/m)	Young	85.1 ± 10.4	64.2 ± 10.4	Very likely ↓
	Middle-aged	69.2 ± 21.1	48.3 ± 9.3	−1.53 (−2.34, −0.71)
20% 1RM peak power (W)	Young	507.9 ± 134.6	486.6 ± 112.7	Very likely ↓
	Middle-aged	387.4 ± 87.9	357.6 ± 86.2	−1.21 (−2.03, −0.39)
80% 1RM peak power (W)	Young	1295.3 ± 369.1	1098.5 ± 307.1	Very likely ↓
	Middle-aged	977.1 ± 211.1	831.9 ± 215.2	−0.94 (−1.76, −0.12)
Blood lactate (mmol·L^−1^)	Young	1.9 ± 0.7	9.8 ± 2.9	Unclear
	Middle-aged	1.6 ± 0.4	8.1 ± 5.2	−0.39 (−1.18, 0.40)

↑ and ↓ denote higher and lower, respectively, in the middle-aged than young group.

**Table 4:** Relationships (qualitative descriptor, upper and lower 90% confidence intervals) of internal and external load markers with fatigue.

Load	Load Markers	MVC	Peak Power	
			20% 1RM	80% 1RM
**Internal**	∆Heart rate	Likely	Unclear	Very likely
		0.45 (0.06, 0.72)	0.28 (−0.14, 0.61)	0.50 (0.13, 0.75)
	Mean OMNI-RPE	Unclear	Likely	Likely
		−0.06 (−0.45, 0.35)	0.36 (−0.05, 0.66)	0.32 (−0.09, 0.64)
	sRPE	Unclear	Unclear	Unclear
		0.07 (−0.34, 0.46)	0.18 (−0.24, 0.54)	0.29 (−0.13, 0.62)
	BLA increase	Unclear	Unclear	Unclear
		0.22 (−0.57, 0.2)	−0.20 (−0.55, 0.22)	−0.19 (−0.55, 0.23)
**External**	Mean peak velocity	Unclear	Unclear	Unclear
		−0.05 (−0.44, 0.36)	0.04 (−0.37, 0.43)	0.02 (−0.38, 0.42)
	Mean peak power	Likely	Likely	Likely
		0.38 (−0.03, 0.68)	0.43 (0.03, 0.71)	0.35 (−0.06, 0.66)
	Volume load	Very likely	Very likely	Very likely
		0.59 (0.24, 0.80)	0.55 (0.19, 0.78)	0.50 (0.13, 0.75)
